# The Congress of American Physicians and Surgeons

**Published:** 1888-08

**Authors:** 


### *THE CONGRESS OF AMERICAN PHYSICIANS AND SURGEONS.*

The first triennial Congress of American Physicians and Surgeons will meet in Washington, September 18, 19 and 20, 1888, under the presidency of Dr. John S. Billings, LL.D., U. S. A.

As it is probable many may not quite understand the scheme of its organization, it would seem proper to briefly refer to it at this time. Some three years ago a proposition was made in the American Surgical Association to group the several American special societies into a congress, with a view to increase the efficiency of their scientific work, and to economize the time of the members, many of whom were in fellowship with several different societies. Attendance upon the meetings of the various societies in such cases required several separate trips from home. It would, therefore, appear that one of the objects to be accomplished was the establishment of a uniform time and place of meeting.

Dr. C. H. Masten, of Mobile, Chairman of the American Surgical Society's Committee, reported at its meeting in 1886, among other things, that the plan involved the following proposition: Each society was to elect its own officers as before; hold its own session apart from the others; publish its own transactions; preserve its own autonomy in every way; the Congress to meet in Washington; the opening session of each meeting to be devoted to such general business as might pertain to the Congress as a whole; the Congress to be presided over by a president, elected at each meeting for the next succeeding term, upon the nomination of a committee of one from each special society in affiliation; the president of each special society to be *ex-officio* a vice-president of the Congress; membership in the Congress to be acquired only by virtue of fellowship in one or other of the special societies.

Dr. Masten's committee recommended that a committee of five be appointed to confer with similar committees from the other special societies, with power to arrange details and to report at the next annual meeting. This was agreed to, and after numerous conferences on the part of the representatives of the societies, it was finally further agreed to organize the Congress upon the following general plan:

The Congress of American Physicians and Surgeons is a conjoint meeting of certain National medical societies, so arranged that, while each society preserves its own autonomy in every respect, and has its own meetings, papers and discussions during the day, the members of all the societies meet together in the evenings to carry out the objects of the Congress.

The main purpose of the Congress is the presentation and discussion of scientific subjects selected with reference to their general interest.

It is intended that the Congress shall meet at Washington, D. C., once in three years. The President of each future Congress is to be elected by the societies, each society having the right to elect in turn.

The officers of the Congress are a President, a Secretary and a Treasurer, and as many Vice-Presidents as there are societies taking part; the Vice-Presidents being the Presidents-elect of the several societies for the year of the Congress.

The business of the Congress is managed by an Executive Committee composed of one representative appointed by each society taking part. The President and Secretary of the Congress are *ex-officio* members of this Committee.

The arrangement of the programme for the Congress of 1888 has been delegated to a Committee consisting of the President of the Congress, the Chairman of the Executive Committee, and the Secretary of the Congress.

Thus the Congress becomes an established fact, and will soon record its work for the judgment of the profession.

It can in no sense appear as the rival of any other National organization, and its friends from the very first have disclaimed any such intention. Particularly the American Medical Association cannot with reason regard it as a rival. The special societies already exist, and the mere grouping of them under one general authority once in two or three years should not—nay, cannot rightfully—invite the jealous criticism of any other medical organization.

The American Medical Association, as we have before said in the columns of the JOURNAL, rests on a foundation peculiarly its own, from which it cannot be driven so long as wise counsels prevail and it performs its work as satisfactorily as at its late meeting in Cincinnati.

We wish the Congress all the success that belongs to conscientious, scientific labor earnestly performed, and we believe that under the guidance of such men as Dr. J. S. Billings as President, Dr. Wm. Pepper, Chairman of the Executive Committee, and Dr. S. C. Busey, Chairman of the Committee of Arrangements, it cannot fail to fulfill the reasonable expectations of its projectors, and leave behind it an additional contribution to the honorable name and fame of American medicine.

